# Protracted exposure to ^134^Cs and ^137^Cs gives substantial contribution to long-term thyroid absorbed dose after nuclear power plant accidents

**DOI:** 10.1093/rpd/ncaf035

**Published:** 2025-08-28

**Authors:** Robert Wålinder, Mats Isaksson, Christopher Rääf, Martin Tondel

**Affiliations:** Occupational and Environmental Medicine, Department of Medical Sciences, University of Uppsala, Akademiska Sjukhuset, 751 85 Uppsala, Sweden; Occupational and Environmental Medicine, Uppsala University Hospital, 751 85 Uppsala, Sweden; Department of Medical Radiation Sciences, Institute of Clinical Sciences, Sahlgrenska Academy, University of Gothenburg, Gula stråket 2B, SU, 413 45 Göteborg, Sweden; Medical Radiation Physics, Department of Translational Medicine, Lund University, Inga Marie Nilssons Gata 47, 205 02 Malmö, Sweden; Occupational and Environmental Medicine, Department of Medical Sciences, University of Uppsala, Akademiska Sjukhuset, 751 85 Uppsala, Sweden; Occupational and Environmental Medicine, Uppsala University Hospital, 751 85 Uppsala, Sweden

## Abstract

Thyroid dose estimations after nuclear power plant (NPP) accidents are traditionally based on internal uptake of radioiodine, mainly ^131^I, either by instrumental measurements of thyroid uptake or by ecological estimations based on geographical dispersion of the radioiodine cloud, demographics, and food habits. However, it has been shown that ^134^Cs and ^137^Cs in some cases can be the dominant contributors to the thyroid dose over long time following NPP accidents. Based on an ecological model using Swedish-specific parameters of the radioactive fallout from the Chernobyl accident in 1986, estimations of the protracted (30 years) thyroid absorbed dose were made for the population in northern Sweden (2.2 million inhabitants in 1986). The internal dose contribution was estimated from both the short-lived nuclides—mainly ^131^I (*T_½,phys_* = 8.1 d) in dairy milk and from inhalation—and nuclides with longer half-lives—^134^Cs (*T_½,phys_* = 2.06 y) and ^137^Cs from aggregate ecological transfer of radiocesium in foodstuff (*T_½,phys_* = 30.2 y). The external radiation dose to the thyroid was based on air-borne measurements of the ground deposition of ^137^Cs, combined with absorbed dose contribution of short-lived radionuclides and with correction for shielding from residential buildings and snow cover. The total thyroid absorbed dose from 1986 to 2015 ranged from 0.06 to 15.5 mGy (mean 2.0 mGy) among subjects in the study population. The calculated mean thyroid absorbed dose the first year was 0.7 mGy, where radioiodine accounted for ~0.3 mGy. The protracted thyroid absorbed dose after 30 years was 0.3 mGy (15%) from ^131^I, and 1.7 mGy (85%) from internal and external ^134^Cs and ^137^Cs taken together. Hence, the estimated mean absorbed dose contribution from radiocesium was higher than for radioiodine (^131^I) both in the first year and in the consecutive 30 years. Furthermore, the 30-year external absorbed dose (1.2 mGy) dominates over the internal absorbed dose (0.8 mGy) to the thyroid. This finding is of relevance for low-dose exposure epidemiological studies of thyroid cancer which previously have focused solely on radioiodine.

## Introduction

The release of Chernobyl nuclear power plant (NPP) debris was widespread, and the local fallout in Europe depended on meteorological conditions such as wind direction and local precipitation. A relatively large proportion of radioactivity reached the Nordic countries. About 5% of the long-lived nuclide ^137^Cs was deposited in northern Sweden, predominantly in the coastal counties along the Bothnian Sea [1].

After the Chernobyl NPP accident direct thyroid gamma measurements were performed in about 400,000 persons in the former USSR [2]. Later dose reconstructions based on these instrumental measurements have shown arithmetic mean thyroid absorbed doses of 0.68 Gy and 0.65 Gy, and maximum absorbed doses of 39 Gy and 42 Gy, in Belarus [3] and in Ukraine [4], respectively. The highest arithmetic mean of 0.96 Gy was found among Pripyat evacuees, ranging from 5.7 mGy to 25 Gy [4]. Gamma measurements within one month after the accident were also performed in Sweden. In Västerbotten County in northern Sweden absorbed doses to the thyroid ranged from 0 to 3.7 mGy, mean 0.37 mGy [5]. The highest thyroid doses in 1986 in Sweden, ranging from 2.7 to 4.4 mGy, were measured on the island Gotland in the Baltic Sea [6].

It is difficult to make direct dosimetry to a large population after a nuclear accident, but algorithms including meteorological dispersion models and fall-out measurements can be used for dose estimations to the population. In a previous study by Rääf *et al.* [7] with dose assessment from fall-out maps in Sweden of ^137^Cs, the iodine cloud shine and inhalation doses, as well as assessments of internal and external exposure from radionuclides during the first 5 years after fallout from the Chernobyl NPP accident showed thyroid absorbed doses ranging from 0.5 to 4.1 mGy for infants and from 0.3 to 3.3 mGy for adults.

The aim of the present study was to apply the same algorithms [7] to estimate the contribution from the Chernobyl NPP accident to the individual thyroid absorbed dose to those 2.2 million people living in middle and northern Sweden. This enables an assessment of the importance of the relative dose contributions from exposure pathways to the thyroid in Sweden that are often disregarded in similar evaluations.

## Material and methods

### Study population

This study was based on a closed cohort of the total population 1986 in nine counties in middle and northern Sweden: Södermanland, Västmanland, Uppsala, Gävleborg, Dalarna, Västernorrland, Jämtland, Västerbotten, and Norrbotten, retrieved from The Register of the Total Population, Statistics Sweden. In 28 April 1986 the cohort comprised 2 156 084 subjects of which there were 734 211 deaths and 77 865 emigrated from Sweden until 31 December 2015. Therefore, the dose assessment does not include individuals born in Sweden after 1986. The absorbed dose assessment is also used in a similar cohort in a current epidemiological study regarding the dose–response analysis on absorbed organ dose and site-specific cancer incidence in Sweden after the Chernobyl NPP accident [8].

### Absorbed thyroid dose estimations

Protracted individual thyroid absorbed doses for the study population from 28 April 1986 to 31 December 2015 were calculated according to the above mentioned model [7]. For a more thorough description of the dose estimation also see [8]. In summary these algorithms take into account the internal absorbed dose contribution from both the short-lived nuclides, mainly ^131^I (with a physical half-life *T_½_* of 8.06 days) and nuclides with longer half-lives, ^134^Cs (*T_½_* = 2.06 years) and ^137^Cs (*T_½_* = 30.2 years). The external radiation absorbed dose to the thyroid was based on air-borne measurements of cesium-activity on the ground with correction for shielding from buildings and snow cover [7, 9]. The time-integrated absorbed dose to the thyroid, *D_th,tot_*, accumulated at a given time after the on-set of fallout, *t*, can then be divided into four components (Eq. 1):


(1) \begin{equation*} {D}_{th, tot}(t)={D}_{milk}(t)+{D}_{inh}(t)+{D}_{ext}(t)+{D}_{cs- ing}(t) \end{equation*}

where D_milk_(t), D_inh_(t) and D_Cs-ing_(t) all three refer to absorbed doses from internal contamination by different exposure pathways, and D_ext_ refers to the external absorbed dose ground deposition of all Chernobyl related radionuclides. *D_milk_*(*t*) is the cumulative absorbed dose contribution from ingestion of ^131^I via dairy milk, *D_inh_*(*t*) is the cumulative absorbed dose contribution through inhalation of airborne ^131^I, *D_ext_*(*t*) is the combined external exposure from ^134,137^Cs and short-lived nuclides on the ground and *D_Cs-ing_*(t) is the cumulative absorbed dose from ingestion of ^134,137^Cs [10].

Absorbed doses were calculated in R program version 4.0.2 [11]. The program code in R is available at https://github.com/absorbedDose/absorbedDose.

## Results

**Figure 1:**
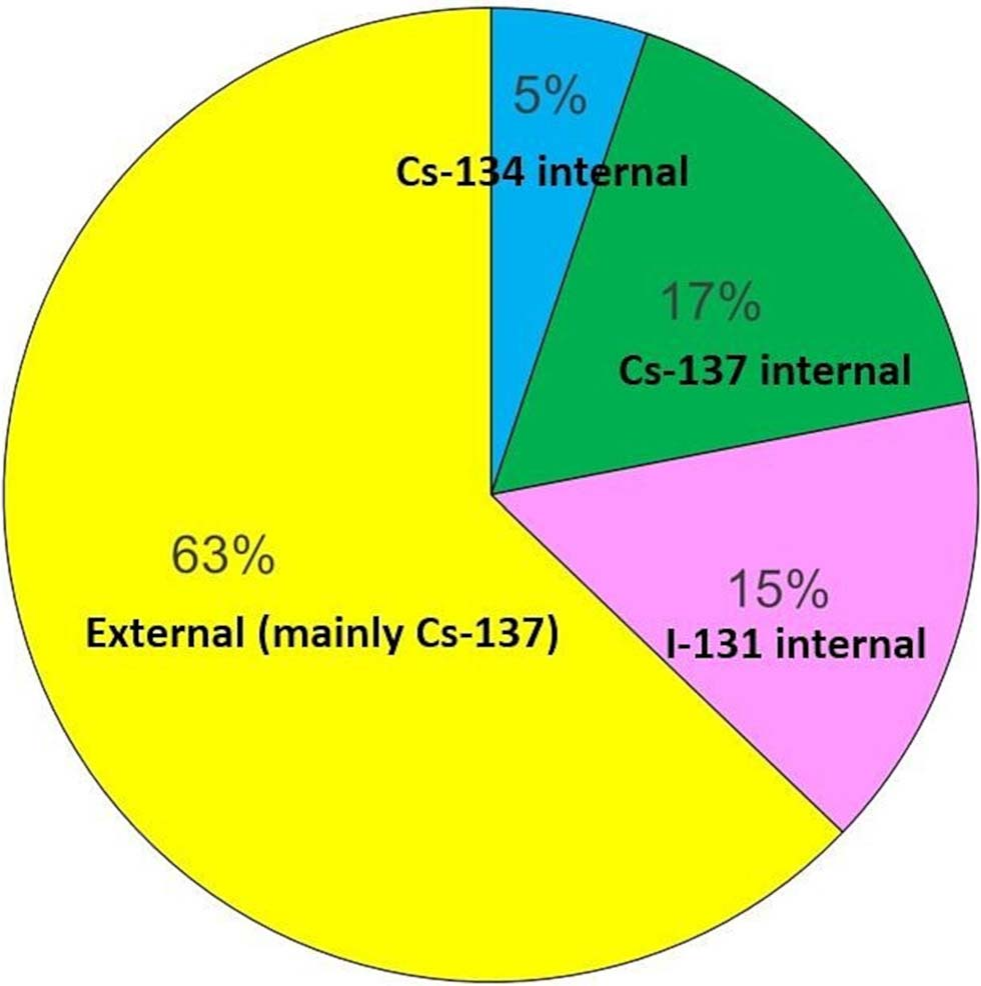
The relative absorbed dose contribution from external (mainly ^137^Cs) and internal absorbed doses to the thyroid from ^131^I, ^134^Cs, and ^137^Cs in the study population from 28 April 1986 to 31 December 2015.

Calculated thyroid absorbed doses from 28 April 1986 to 31 December 2015 (*D_th,tot_* in Eq. 1) ranged from 0.06 to 15.5 mGy, with arithmetic mean absorbed doses of 2.0 mGy (Table 1). The first year the total mean thyroid absorbed dose was 0.7 mGy with a contribution from ^131^I of 0.29 mGy (41%). For the whole study period of 30 years the contribution from ^131^I was 15% (Fig. 1), whereas the external absorbed dose contribution from ^134^Cs and ^134^Cs over 30 years was 1.2 mGy (63%). An additional 0.8 mGy thyroid absorbed dose emanates from internal radiation from radioiodine and radiocesium (Table 1).

**Table 1:** Calculated absorbed doses during 30 years to the thyroid gland in the population (2.2 million) living in middle and northern Sweden 28 April 1986 from inhalation, ingestion and external radiation from the radionuclides ^131^I, ^134^Cs and ^137^Cs.

	28 April 1986 to 31 December 2015							
	Female thyroid absorbed dose (mGy)				Male thyroid absorbed dose (mGy)			
Nuclide	Mean	Median	5–95th perc	Min–max	Mean	Median	5–95th perc	Min–max
I-131 internal	0.310	0.349	0.056–0.866	0.056–1.078	0.312	0.349	0.056–0.874	0.056–1.078
Cs-134 internal	0.078	0.073	0.014–0.185	0.000–0.717	0.123	0.122	0.022–0.317	0.000–1.125
Cs-137 internal	0.288	0.242	0.043–0.806	0.000–3.322	0.472	0.362	0.065–1.350	0.000–5.576
Total external	1.259	0.663	0.172–3.954	0.000–14.718	1.179	0.609	0.159–3.748	0.000–13.535
Total (int + ext)	1.935	1.415	0.321–4.992	0.056–15.545	2.086	1.542	0.354–5.363	0.056–15.542

**Figure 2:**
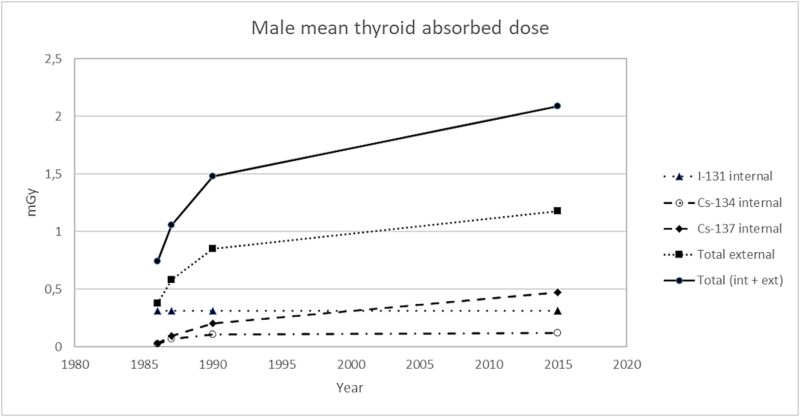
Cumulative absorbed doses to males (~1.1 million) who were living in middle and northern Sweden 28 April 1986 and 30 years forward, showing graphs of total, internal (^131^I, ^134^Cs, and ^137^Cs), and external (mainly ^137^Cs) radiation doses.

**Figure 3:**
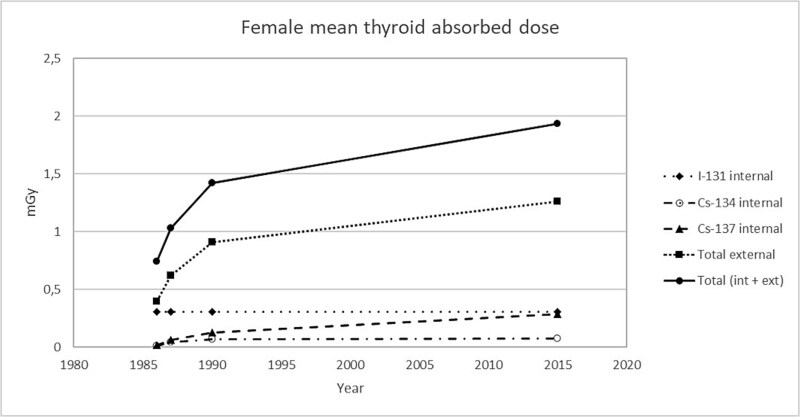
Cumulative absorbed doses to women (~1.1 million) who were living in middle and northern Sweden 28 April 1986 and 30 years forward, showing graphs of total, internal (^131^I, ^134^Cs, and ^137^Cs), and external (mainly ^137^Cs) radiation doses.

Furthermore, there appears to be little difference between the sexes in the thyroid absorbed dose from the Chernobyl fallout in in Sweden (Figs. 2 and 3). However, there is a slightly higher relative contribution among males from ^137^Cs and ^134^Cs, being an effect of males having, on average, higher body concentrations (Bq kg^−1^) of radiocesium than females among populations living in the same region [10].

## Discussion

Calculated absorbed doses to the thyroid were low compared with all previous population studies that have shown increased risk of thyroid cancer from the Chernobyl NPP accident. Previous thyroid scans after the Chernobyl NPP accident in Belarus and Ukraine had mean absorbed doses to the affected populations above 600 mGy. These absorbed dose estimates were based on about 400,000 thyroid scans of ^131^I between 26 April and 30 June 1986 [12]. Though estimated absorbed doses of the present study were low, they are in agreement with a direct thyroid scan survey of ^131^I activity made in northern Sweden directly after the accident in 1986 showing a mean thyroid absorbed dose the first month of 0.4 mGy [5]. The cloud of radioiodine might have given higher exposure to ^131^I in southern Sweden, which are populations not included in the study cohort, but as the main fallout from precipitation with long-lived nuclides was higher in northern Sweden the average individual total absorbed thyroid doses were higher in north Sweden. Most post-Chernobyl studies, with the exception of some few studies [2,6,13,14], only include radioiodine in the thyroid dose model [3, 15–18].

^131^I contributed to about 90% of the thyroid absorbed dose for populations living close to the Chernobyl NPP [4], but in the present study with a dose model including long-lived radiocesium the dose contribution from radiocesium exceeded that for radioiodine already the first year. This is also in agreement with a study on evacuees who lived close to the Chernobyl NPP, showing that other nuclides than ^131^I and external radiation could account for about 40% of the thyroid absorbed dose [19]. One reason for the lower radioiodine contribution to the absorbed dose to the thyroid could be countermeasures such as grazing restrictions and control of dairy milk were done in Sweden already within days after the fallout [20]. Whereas in the areas close the Chernobyl fallout many hundred thousands of individuals were unknowingly exposed to radioiodine intakes through inhalation of the passing radioactive cloud and through intakes of radioiodine contaminated dairy milk [21]. Nevertheless, this study can give a notion on which exposure pathways to the thyroid that can be of radiological relevance in case when initial emergency countermeasures are undertaken to avert transfer through pasture and dairy milk to man.

## Conclusions

This study specifically evaluated the relative absorbed dose contributions from different nuclides to the thyroid in populations exposed to the Chernobyl NPP fallout in Sweden after 1986. Radiocesium isotopes are far more long-lived than those of radioiodine and can therefore dominate the absorbed dose to the thyroid already after one year at larger distances from a NPP accident. Results could also to some part reflect the outcome of early preventive measures on grazing and dairy milk in Sweden in 1986 that reduced the radioiodine transfer to humans. The model presented here could be used for scenarios at a similar distance from a NPP accident for dose estimations and the basis for correct countermeasures.

## CRediT statement

Robert Wålinder: Writing original draft, review and editing. Mats Isaksson, Christopher Rääf and Martin Tondel: Methodology, validation of results, review and editing.
